# Relationship between Oscillatory Neuronal Activity during Reward Processing and Trait Impulsivity and Sensation Seeking

**DOI:** 10.1371/journal.pone.0083414

**Published:** 2013-12-20

**Authors:** Gregor Leicht, Stefan Troschütz, Christina Andreou, Evangelos Karamatskos, Matthias Ertl, Dieter Naber, Christoph Mulert

**Affiliations:** 1 Department of Psychiatry and Psychotherapy, Psychiatry Neuroimaging Branch (PNB), University Medical Center Hamburg-Eppendorf, Hamburg, Germany; 2 Department of Neurology, Ludwig-Maximilians-University, Munich, Germany; 3 Graduate school of systemic neuroscience, Ludwig-Maximilians-University, Munich, Germany; University College of London - Institute of Neurology, United Kingdom

## Abstract

**Background:**

The processing of reward and punishment stimuli in humans appears to involve brain oscillatory activity of several frequencies, probably each with a distinct function. The exact nature of associations of these electrophysiological measures with impulsive or risk-seeking personality traits is not completely clear. Thus, the aim of the present study was to investigate event-related oscillatory activity during reward processing across a wide spectrum of frequencies, and its associations with impulsivity and sensation seeking in healthy subjects.

**Methods:**

During recording of a 32-channel EEG 22 healthy volunteers were characterized with the Barratt Impulsiveness and the Sensation Seeking Scale and performed a computerized two-choice gambling task comprising different feedback options with positive vs. negative valence (gain or loss) and high or low magnitude (5 vs. 25 points).

**Results:**

We observed greater increases of amplitudes of the feedback-related negativity and of activity in the theta, alpha and low-beta frequency range following loss feedback and, in contrast, greater increase of activity in the high-beta frequency range following gain feedback. Significant magnitude effects were observed for theta and delta oscillations, indicating greater amplitudes upon feedback concerning large stakes. The theta amplitude changes during loss were negatively correlated with motor impulsivity scores, whereas alpha and low-beta increase upon loss and high-beta increase upon gain were positively correlated with various dimensions of sensation seeking.

**Conclusions:**

The findings suggest that the processing of feedback information involves several distinct processes, which are subserved by oscillations of different frequencies and are associated with different personality traits.

## Introduction

The ability to evaluate the outcomes of one's actions is of cardinal importance for learning and decision-making, and ultimately for developing adaptive behaviors. This function is carried out by the reward network, which comprises several frontostriatal and midbrain areas [1], [2], [3]. Insights into the mechanisms involved in the propagation and integration of information across this extensive network are important for our understanding of normal behavior as well as psychiatric disorders such as psychotic, mood and substance disorders. With its excellent temporal resolution, electroencephalography provides an ideal means of investigating the dynamics of the above network.

Event-related potential studies investigating the processing of positive (reward) or negative (punishment) feedback in gambling tasks have identified a negative deflection that reaches its maximal amplitude 250 to 300 ms following negative feedback stimuli, the so-called feedback- (or outcome-) related negativity (FRN) [4], [5], [6]. The latter belongs to the general family of medial frontal negativities (MFN), i.e. event-related potentials with a frontal scalp distribution elicited by error responses or feedback related therewith [7]. The FRN is thought to represent the output of a cognitive system that indicates whether goals (previously established depending on the task context) have been satisfied [4].

It has been demonstrated that the FRN is mainly composed of oscillations in the theta frequency range [6], [7], [8], [9], whereby negative feedback is associated with an increase in theta power. In contrast, positive events are associated with increase of power in the high beta [6], [8] and/or low gamma frequency band [10] about 200–400 ms after feedback (although see also [11], [12]). In contrast to the theta response, which appears to distinguish feedback stimuli in a binary manner, i.e. ‘good’ vs. ‘not good’ (the latter subsuming both neutral and negative events) [4], [6], there is evidence that the beta power increase after rewarding events might be modulated both by their probability and their magnitude [6], [8], [10]. Since different oscillatory frequencies have been suggested to represent the activity of different processes/networks [13], [14], the above dissociation implies the existence of two distinct neural networks subserving processing of positive and negative feedback stimuli (cf. [6]). This notion is supported by fMRI studies on reward [15], [16].

However, little is known about how these electrophysiological markers relate to behaviors and personality traits associated with the reward system, such as risk-seeking behavior or impulsivity (e.g. [17]). A number of previous studies investigating task-related risk-taking or impulsive behavior have reported mixed results regarding its correlation with FRN amplitudes or theta power [5], [10], [18]. However, task-related behavior is not necessarily a marker of trait impulsivity or sensation-seeking: It has been demonstrated that risk-seeking behavior during gambling tasks is modulated by the recent history of gains and losses in healthy subjects, increasing linearly with the magnitude of losses [19]. Thus, measures of task-related impulsivity are quite likely to be affected at least as much by chance (i.e. the sequence of winning and losing trials) as by personality characteristics. Regarding the latter, so far few studies have investigated the association between impulsive personality traits and electrophysiological responses to feedback. It is suggested that impulsive individuals exhibit diminished reactivity of the reward system, reflected in reduced FRN and/or theta oscillatory responses [12], [20], [21]. However, the evidence for this hypothesis is so far rather weak, and findings have not always been replicated [18]. Moreover, it is not known whether the assumed reward deficiency applies only to loss-related theta oscillations, or also to reward-related beta/gamma oscillations. This question is relevant in the face of an earlier account of impulsivity that postulates the existence of two separate systems subserving reward and punishment, an imbalance of which results in diminished responsivity to loss but also increased responsivity to reward [22]. Since recent evidence indeed supports the existence of two distinct reward systems (see above) it would be important to examine whether both of these are dysfunctional in impulsive individuals.

It should be pointed out here that theta and beta are not the only oscillatory frequencies implicated in reward system functions. Alpha power changes in the human ventral striatum have been implicated in reward learning [23], [24], and the magnitude of frontal alpha power asymmetry has been shown to predict behavior during reward tasks [25], [26], [27]. Delta oscillations have also been implicated in the reward functions of drugs of abuse, as well as in withdrawal and craving symptoms during abstinence (for a review, see [14]), and have been suggested to play an important role in anticipation and motivation [28], [29].

In summary, the processing of reward and punishment stimuli in humans appears to involve several band frequency oscillations, probably each with a distinct function. Although there is evidence for an association of electrophysiological responses to feedback with impulsive or risk-seeking personality traits and behaviors, the exact nature of this association is not completely clear. Thus, the aim of the present study was to investigate event-related oscillatory activity during feedback processing across a wide spectrum of frequencies, and its associations with impulsivity and sensation seeking in healthy subjects.

## Methods

### Ethics Statement

The study was approved by the Ethics Committee of the Medical Association Hamburg and written informed consent according to the guidelines of the Declaration of Helsinki was obtained from all participants.

### Study Design

**Figure 1:**
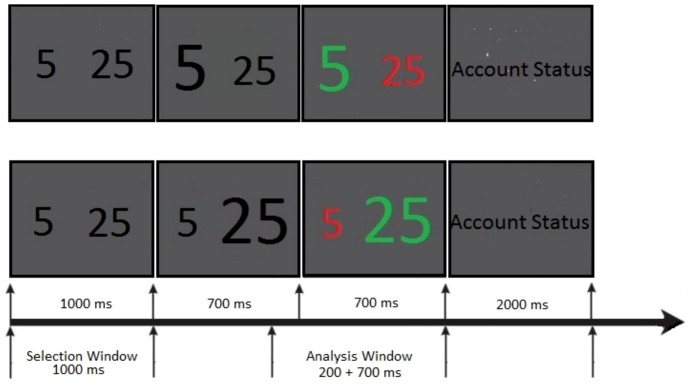
Paradigm. Schematic diagram showing the design of a trial of the gambling task used in this study.

22 right-handed healthy volunteers (16 female, 6 male, mean age 26.3±3.1 years) with no history of neurological or psychiatric disturbance were recruited from an academic environment of the University of Hamburg. Participants performed a computerized two-choice gambling task [19], which had been used in similar EEG investigations before [5], [6]. The task involved a low-risk and a high-risk option. On each trial two numbers (5 and 25) were presented in black color on a grey background in the middle of a computer screen as two possible displays: either [25] [5] or [5] [25] in a randomized order (see Fig. 1). The visual angle of stimuli was 1.15°. Participants were asked to select one of the presented numbers within one second after stimulus onset, by pressing the right or left mouse button to indicate selection of the number presented on the right (e.g. 5 in the [25] [5] display) or left side of the screen, respectively. If the participants did not respond within the allowed time, the trial was considered as an error and was terminated. One second after stimulus onset the font weight of the selected number was set to bold. After a further delay of 700 ms one of the two numbers randomly turned green while the other one changed its color to red (feedback stimulus). This color change represented the feedback that indicated whether the participant had earned a gain or lost: if the number selected by the participant (font weight: bold) turned green, this symbolized a gain of the corresponding amount of points; a color change to red indicated a respective loss of points. Every participant started the experiment with an amount of 1000 points. After presentation of the gain/loss feedback, the current account status was presented for 2 seconds. The trial ended with a 3-second interval, during which a fixation square was present on the screen.

The experiment comprised two blocks of 86 trials each. The occurrence of loss and gain conditions was maintained at equal probability (50% each). For the analysis, 4 different feedback conditions were defined irrespectively of left or right choices: maximum gain (participant gained +25 points), minimum gain (+5 points), maximum loss (participant lost −25 points) and minimum loss (−5 points). For example, if a participant chose 25 in a [**25**] [5] or a [5] [**25**] event (bold stands for green  =  win), the trial was counted as a maximum gain trial, whereas the selection of 5 in the given examples was counted as a minimum loss trial. Gained or lost points were added to or subtracted from the account status, respectively. Before starting a practice block and the EEG recordings, all participants were instructed in a standardized way to freely choose one of the two presented numbers (5 or 25) in every trial and to gain as many points as possible during the experiment. The paradigm was created and presented with the Presentation software (version 14.4).

### EEG Recording

Recording took place in a sound-attenuated and electrically shielded room. Subjects were seated with open eyes in a slightly reclined chair with a head rest and were asked to look at the 19″ computer monitor 1 m in front of them. The EEG was recorded with 32 active electrodes mounted on an elastic cap (ActiCaps, Brain Products, Munich, Germany). 19 electrodes were positioned according to the International 10/20 system and 13 additional electrodes (FC1, FC2, FC5, FC6, CP1, CP2, CP5, CP6, PO9, PO10, TP9, TP10 and Oz) were positioned in between. An electrode at the FCz position served as the reference electrode, AFz served as ground. Electrode impedances were always kept below 5 kΩ. The EEG recording was acquired using the Brain Vision Recorder software Version 1.10 (Brain Products, Munich, Germany). Data were collected with a sampling rate of 1000 samples per second.

### EEG pre-processing

Data analysis was done using the Brain Vision Analyzer software Version 2.0 (Brain Products, Munich, Germany). After band-pass filtering (0.1–100 Hz), all data sets were corrected for eye-blink artifacts by applying an ICA. The continuous EEG was segmented into epochs of 3 seconds starting 1800 ms prior to the feedback stimulus (color-change of the number display). Segments including amplitudes exceeding ±95 µV, voltage steps higher than 50 µV between sampling points, a difference between a segment's highest and lowest value higher than 200 µV or activity below 0.5 µV were automatically rejected. After re-referencing to common average reference, a baseline correction (using an interval of 200 ms prestimulus) was applied. Averaged ERP wave-shapes were computed within each subject and condition with a minimum number of 20 trials per condition. Two subjects (1 female, 1 male) had to be excluded due to a number of trials less than 20 in one of the four conditions. The mean number of trials included in the averages was 36.3 (SD = 8.7). The number of trials included in the averages did not significantly differ across conditions. For presentation of results, data were segmented into epochs with a duration of 900 ms starting 200 ms prior to the feedback stimulus after averaging.

### FRN

**Figure 2:**
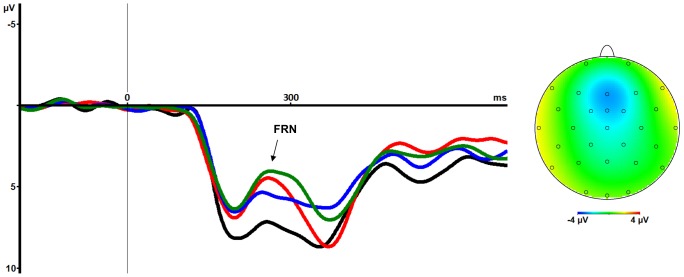
Feedback related negativity. Grand average waveforms of feedback-related visual evoked potentials for the maximum loss (red), maximum gain (black), minimum loss (green) and minimum gain (blue) condition showing the FRN effect with larger FRN amplitudes in response to loss feedback compared to gain feedback (onset of feedback stimuli at 0 ms). The scalp topography (derived from the peak amplitude of the difference waveform of maximum loss and maximum gain condition observed 270 ms after presentation of the feedback stimulus) shows a frontocentral maximum over Fz.

The FRN-amplitude was defined as the peak-to-peak distance between the negative local maximum value within the timeframe 220–300 ms after the feedback stimulus (±40 ms from the FRN peaking around 260 ms poststimulus in the averages over all subjects, see Fig. 2) and the preceding positivity defined as most positive value within the timeframe 180–220 ms poststimulus (±20 ms from the observed latency of this peak around 200 ms poststimulus, see Fig. 2) at electrode Fz.

### Time-frequency analysis

**Figure 3:**
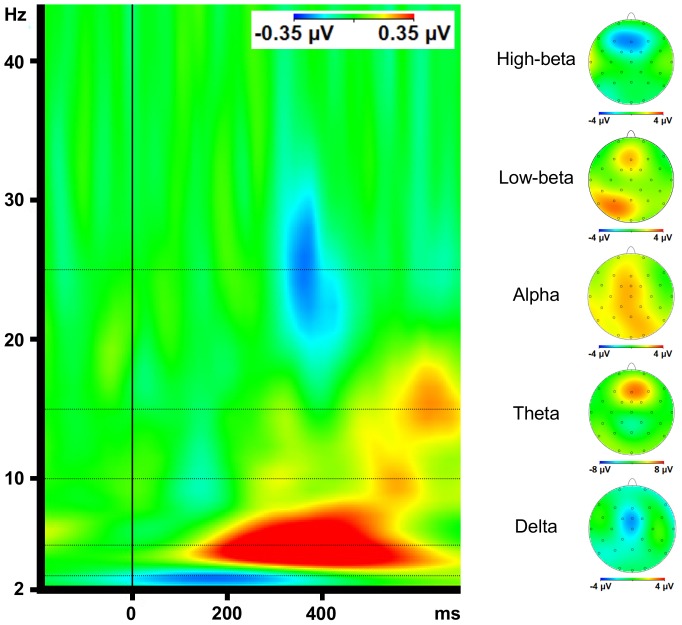
Time frequency analysis. The comparison (difference) of the results of the time-frequency analysis of maximum loss and maximum gain conditions revealed theta, alpha and low-beta activity to be more pronounced in the maximum loss condition and delta and high-beta activity to be more pronounced in the maximum gain condition (onset of feedback stimuli at 0 ms). For all frequencies, we found frontocentral maxima of differences between conditions. The scalp topographies are derived from the peak amplitudes of the difference waveforms (maximum loss minus maximum gain condition) of the extracted frequency-specific wavelet layers (latencies: delta 150 ms; theta 340 ms; alpha 550 ms; low-beta 630 ms; high-beta 360 ms). The dotted lines indicate the frequencies of interest (from the bottom up: delta, theta, alpha, low-beta and high-beta).

Before averaging, a continuous wavelet transformation using a complex morlet wavelet (formula: w(t)  =  Aexp(−t^2^/2)exp(i2πct), 50 frequency steps distributed on a logarithmic scale, Morlet parameter c = 5, Gabor Normalization) was calculated for the frequencies from 2 to 80 Hz for every segment. Complex morlet wavelets have frequently been used for time-frequency analysis by ours [30], [31] and other laboratories [32]. The results of the wavelet transformation were averaged within each subject and condition and grand averages containing all 20 subjects were calculated. To compare differences between conditions with respect to activity in different frequency ranges, we extracted wavelet layers with central frequencies of 3 (delta), 5 (theta), 10 (alpha), 15 (low-beta) and 25 Hz (high-beta) from our wavelet analysis for every subject and condition. The peak-amplitudes of activity in these frequencies of interest were defined as the highest values within the timeframes 0–700 ms poststimulus for delta, 100–700 ms poststimulus for theta, 550–700 ms poststimulus for low-beta and 200–500 ms poststimulus for high-beta. Alpha-activity was parameterized as the mean amplitude in the timeframe between 500 and 600 ms poststimulus. The frequencies of interest and the time frames for peak detection were defined according to the results of the comparison (difference) of maximum loss and maximum gain conditions (see Fig. 3).

### Measures of impulsivity and sensation seeking

Trait impulsivity was assessed with the Barratt Impulsiveness Scale (BIS), a 30-item Likert-type self-report questionnaire [33] yielding scores for attentional, motor and non-planning impulsivity. The BIS has been widely used in similar studies and has good reliability and validity [33], [34].

Sensation seeking was assessed by means of the Sensation Seeking Scale (SSS). The latter consists of 40 forced-choice items. Participants' responses are used to calculate four subscores: thrill and adventure seeking, disinhibition, experience seeking, and boredom susceptibility [35].

### Statistical Analysis

All statistical analyses were performed using the SPSS software package (21.0). A 2 (valence: positive vs. negative feedback) ×2 (magnitude: 5 vs. 25 points) repeated-measures ANOVA was conducted for each variable of interest. Based on the results of these ANOVAs, difference scores reflecting significant main effects in each frequency band were calculated (e.g., in the case of a significant valence effect, this was operationalized as the difference score between mean amplitude in the positive minus mean amplitude in the negative feedback condition). Correlational analyses were then conducted between these difference scores and BIS/SSS subscales. As most variables were normally distributed, these analyses were conducted using Pearson's r. Bootstrapping was applied to calculate confidence intervals of correlation coefficients. Note that correlational analysis results are based on 15 subjects, as not all subjects completed the self-rated questionnaires.

## Results

Characteristics of the participant group and BIS/SSS scores are presented on Table 1. The mean loss of the participants after performing the 172 trials was 17.5±206 points. 10 participants ended the experiment gaining some points, 12 participants lost points. Mean values per condition for all electrophysiological measures of interest, as well as ANOVA results are presented on Table 2.

**Table 1:** Characteristics of the participant group.

	N	
Gender (m/f)	5/15	
	Mean	SD
Age	26.25	3.09
Barratt Impulsiveness Scale	59.73	5.56
attentional	13.67	1.76
motor	21.80	3.30
non-planning	24.13	3.07
Sensation Seeking Scale	22.80	5.12
thrill and adventure seeking	6.60	2.59
disinhibition	5.47	1.81
experience seeking	6.20	1.57
boredom susceptibility	3.93	2.19

**Table 2:** Mean values of electrophysiological measures per condition and ANOVA results.

									ANOVA results					
	Maximum Gain		Minimum Gain		Maximum Loss		Minimum Loss		valence		magnitude		valence × magnitude	
	*Mean*	*SD*	*Mean*	*SD*	*Mean*	*SD*	*Mean*	*SD*	*F(1,19)*	*p*	*F(1,19)*	*p*	*F(1,19)*	*p*
FRN (amplitude)	3.72	2.34	3.61	2.02	4.97	2.61	4.72	2.24	15.15	**0.001**	0.58	0.46	0.09	0.77
FRN (latency)	260	24	262	24	263	22	261	23		n.s.		n.s.		n.s.
Delta (amplitude)	2.22	0.40	2.07	0.37	2.19	0.50	2.13	0.48	0.68	0.80	9.29	**0.01**	1.53	0.23
Delta (latency)	288	145	315	147	398	154	395	166		n.s.		n.s.		n.s.
Theta (amplitude)	2.70	0.67	2.58	0.65	3.31	0.91	3.09	0.93	18.15	**<0.001**	9.74	**0.01**	0.87	0.36
Theta (latency)	324	107	319	137	337	75	344	92		n.s.		n.s.		n.s.
Alpha (amplitude)	2.10	1.00	2.21	0.90	2.27	0.96	2.32	1.01	7.15	**0.02**	2.71	0.12	0.28	0.60
Low-beta (amplitude)	1.74	0.49	1.84	0.44	2.02	0.44	1.78	0.47	5.52	**0.03**	2.71	0.12	10.54	**0.004**
Low-beta (latency)	639	41	631	40	642	37	633	49		n.s.		n.s.		n.s.
High-beta (amplitude)	1.88	0.63	1.95	0.61	1.32	0.27	1.36	0.30	33.31	**<0.001**	4.64	**0.04**	0.13	0.72
High-beta (latency)	385	80	343	99	344	71	369	86		n.s.		n.s.		n.s.

Amplitudes are reported in µV; latencies are reported in milliseconds. n.s.  =  not significant.

A FRN with maximum peak at frontocentral sites and around 260 ms was observed in the grand averages of all conditions (see Fig. 2). A significant valence effect was observed, i.e. FRN amplitude was larger following negative compared to positive feedback. Neither the main effect of magnitude, nor the valence × magnitude interaction were significant. There was no significant ANOVA effect with respect to the latency of the FRN.

The comparison (difference) of the results of the time-frequency analysis of maximum loss and maximum gain conditions is presented in Fig. 3. Significant valence effects were noted for theta, alpha and high-beta activity, indicating greater increase of amplitudes following loss compared to gain in the theta and alpha frequency range and, in contrast, greater increase of amplitudes following gain compared to loss in the high-beta frequency range. With respect to low-beta activity, a significant valence effect was qualified by a significant valence × magnitude interaction reflecting amplitude increase only in the maximum loss condition (i.e. loss of 25 points).

Significant magnitude effects were observed for theta and delta oscillations, indicating greater amplitudes upon feedback concerning large stakes (25 points) compared to small stakes (5 points). Moreover, a significant magnitude effect was found for high-beta activity, but in the opposite direction.

No significant ANOVA effects on the latencies of delta, theta, low-beta and high-beta peaks were observed except a significant valence effect on latency for the delta frequency range [F(1,19) = 8.09, p = 0.01].

Correlational analyses showed a significant negative correlation between theta valence difference score and BIS motor impulsivity (r = .610, p = 0.02, 95% CI = .222–.822), i.e., the greater the theta amplitude increase upon loss, the lower the score on the motor subscale of the BIS. On the other hand, SSS thrill and adventure seeking and SSS experience seeking exhibited significant positive correlations with alpha (r = .755, p = 0.001, 95% CI = .403–.905) and low-beta (r = .615, p = 0.02, 95% CI = .149–.905) valence difference scores respectively, i.e. greater increase of alpha and low-beta amplitudes upon loss was associated with higher scores on the respective SSS subscales. Delta magnitude difference scores were positively correlated both with BIS motor impulsivity (r = .577, p = 0.02 CI = .125–.820) and with SSS disinhibition, although the latter correlation showed large variability (r = .536, p = 0.04 CI =  0.036–0.865).

## Discussion

The present study investigated oscillatory responses associated with gain and loss in healthy subjects during a gambling task. A differential pattern was observed for different frequency bands: Theta, alpha and low-beta amplitudes increased after loss events, while beta oscillations increased after gain events, and delta oscillations were affected only by the magnitude of feedback irrespectively of its valence. The observed theta amplitude changes during loss were negatively correlated with motor impulsivity scores on the BIS, whereas alpha and low-beta increase upon loss and high-beta increase upon gain were positively correlated with various dimensions of sensation seeking. These findings suggest distinct roles of various frequency band oscillations in the integration of information across the reward system.

Regarding FRN and theta findings, our results are largely in accordance with previous literature. FRN amplitude was higher in loss compared to gain trials but was not affected by reward magnitude, similar to previous studies [4], [6], [25], [36], [37], [38], [39], [40]. Similarly, negative feedback was associated with increased theta amplitude, consistent with previous studies [6], [7], [8], [9], [10] and with the assumption that theta constitutes the major component frequency of the FRN [6], [7], [8], [9]. Moreover, theta oscillations have been found to be related to reward processing in animal studies [41], [42]. Regarding the effect of feedback magnitude on theta amplitudes, only two studies have so far investigated this issue. In one of these, this effect was significant [10], while in the other it was not [6]. Differences in statistical analysis methods preclude strong inferences regarding the nature of this discrepancy; however, in the latter study [6], there was some evidence of FRN modulation by feedback magnitude. Thus, it might be that theta oscillations do not only encode outcome valence, but also its magnitude. Alternatively, it is possible that the magnitude effect observed in the present study was due to frequency smoothing from the delta frequency range, which also showed a significant effect of magnitude (see below).

In the beta frequency range, greater increase in amplitudes upon positive compared to negative feedback has been reported previously for oscillatory components in the high-beta to low-gamma frequency range [6], [8], [10], a finding we were able to reproduce in the present study. In contrast, other studies have reported a larger beta oscillatory response in loss rather than reward [11], [12]. It has been argued that the variability in findings regarding beta responses to reward might be related to differences in task demands [11]. So far it is not clear what these differences might be. However, close inspection of the experimental paradigms used in the above studies indicates that the time between participant response and feedback was shorter in studies reporting significant increase of beta/gamma oscillations with reward than in those reporting a decrease (up to 1 sec vs. ≥1.4 sec). Thus, it is conceivable that the anticipatory period might modulate high-beta responses to reward, by e.g. affecting the emotional salience of the feedback stimulus (cf. [10]). This interpretation is purely speculative at this point; further studies that directly investigate this issue are warranted.

It appears then that there is indeed a dissociation among the responses of different frequency oscillations to feedback events, with the power of lower frequency oscillations increasing upon loss, whereas high frequency oscillations might respond preferentially to gain, at least in some circumstances. This, in turn, might imply that different frequency oscillations have differential functions within the reward system, consistent with the notion of two separate neural networks for the processing of positive and negative feedback (see Introduction). Interestingly, power increase upon negative feedback was not only observed for theta in the present study, but also in the middle frequency range (alpha and low-beta). This is not a surprising finding, since changes in alpha activity have been observed in the human ventral stiatum/nucleus accumbens during reward learning, and have been postulated to mediate synchronization of this area with other parts of the reward network, mainly the orbitofrontal and prefrontal cortex [23], [24]. Further, our result replicates a previous report of alpha power increase following losses during a competitive decision-making task [11]. What is still unclear is the functional role of this activity. Alpha activity has been often ascribed an inhibitory role [43]. However, one study reported increase of alpha power in the nucleus accumbens during a gambling task [23], suggesting thus a different functional role of alpha oscillations within the context of reward tasks. Tentative evidence suggests that this role might consist in coordinating the timing of firing of functionally dissociable, but spatially overlapping neural populations, thus enabling the reward network to differentiate its activity during gains vs. losses [23]. This is consistent with evidence implicating alpha oscillations in top-down and attentional processing, whereby alpha can both reflect processing in task-relevant networks or inhibition of task-irrelevant regions [44], [45].

In contrast to all other frequencies, delta power did not appear to be affected by feedback valence, but only by its magnitude. This finding is interesting, since it might explain the widely discrepant findings regarding delta activity in patients with substance disorders (for a review of these findings, see [14]). Relevant for the interpretation of this magnitude effect are studies linking delta oscillatory activity to the P3 potential, an event-related potential that appears as a response to rare or salient events that require a discriminative decision or motoric response. Delta oscillations have been shown to constitute the most predominant component of the P3 potential [46], [47], [48], [49], and a recent review has linked enhanced P3-related delta activity to the motivational relevance of the task and the salience of the target stimulus [28]. The magnitude effects observed in the present study might thus reflect the enhanced salience and motivational impact of feedback regarding large stakes, irrespectively of its valence. In this context, delta oscillations might serve to adjust future decision making depending on the history of previous feedback, since it has been shown that oscillations in this frequency range can modulate attentional selection during evidence gathering [50], [51].

Various indices of oscillatory response to feedback were associated with impulsive and sensation-seeking personality traits. Consistent with previous findings [12], [21], theta amplitude increase upon negative feedback correlated negatively with motor impulsivity scores. This finding is in line with the hypothesis of reward deficiency syndrome [52] at the basis of impulsive personality. In this context, our finding of positive correlation between delta magnitude difference score and motor impulsivity could be interpreted as reflecting a lower motivational impact of small stakes in impulsive individuals. On the other hand, the magnitude of high-beta response to reward did not correlate with any dimension of impulsivity. This clearly refutes the assumption of increased reward responsiveness in impulsive individuals [22]. A recent functional neuroimaging study in patients with impulsivity-related personality disorders [53] also found reduced activation of prefrontal and other brain areas in the context of both reward and loss in patients compared to healthy controls, supporting the assumption of a reward deficiency syndrome. Thus, our results provide at least partial support for the reward deficiency hypothesis. We did not, however, find evidence of reduced beta reactivity in more impulsive individuals. There might be several reasons for this: For example, it is possible that reward reactivity encompasses aspects other than high-beta oscillations, which are more adequately captured by functional MRI. Alternatively, our negative finding might be simply due to the small sample size available for correlational analyses, or to relatively low variance of impulsivity scores in our healthy participants compared to patients. Further studies with larger sample sizes are needed to clarify this issue.

Interestingly, sensation seeking showed a different pattern of correlations with feedback-related oscillations than that of impulsivity. This observation confirms the view that sensation seeking and impulsivity represent distinct, though correlated, personality traits [54]. In the present study, thrill-seeking and experience seeking were positively correlated with the magnitude of alpha and low-beta response to loss. As already mentioned above for alpha, oscillations in the middle range of the frequency spectrum appear to be involved in mechanisms of top-down modulation and higher cognitive processes, such as attention and working memory [44], [45]. It is conceivable, then, that enhanced alpha and low-beta reactivity to loss in high sensation-seeking individuals reflects a disturbance in the processing of negative feedback events, requiring larger computational resources for their integration. For example, beta oscillations have been proposed to signal an effort to maintain the current cognitive set in the face of unexpected external events [44]. According to this account, our findings could indicate that sensation seeking individuals exhibit a positive bias in their expectations, reacting to negative feedback as an unexpected event. Given that behavioral and observational studies have indeed found an “optimism bias” to promote risk-taking behavior [55], [56], [57], this assumption would be interesting to investigate in further studies. However, it should be noted her that interpretation of correlational patterns is limited by the small sample size. Although the robust confidence intervals obtained with bootstrapping lend credibility to the significant results, the sample size was too small to correct for multiple testing appropriately. Moreover, a larger sample size might have revealed a richer pattern of correlations.

In conclusion, the findings of the present study suggest that the processing of feedback information involves several distinct processes, which are subserved by oscillations of different frequencies and are associated with different personality traits.
